# The impact of parenting style on malevolent creativity based on Chinese university students: a latent profile analysis

**DOI:** 10.3389/fpsyg.2024.1363778

**Published:** 2024-06-26

**Authors:** Xingnan Cui, Xiang Zhang, Hua Zhang

**Affiliations:** ^1^Faculty of Psychology, Southwest University, Chongqing, China; ^2^School of Mechanical Engineering, Southwest Jiaotong University, Chengdu, China

**Keywords:** latent profile analysis, malevolent creativity, moral, parenting style, university student

## Abstract

**Introduction:**

This study investigated the association between parenting styles and malevolent creativity.

**Methods:**

It used latent profile analysis to compare the differences in malevolent creativity between different combinations of parenting styles with an online sample (*N* = 620).

**Results:**

The results of the study suggest that a three-profile solution best fits the data, and the three profiles were labelled *positive open parenting*, *undifferentiated parenting* and *negative limited parenting*. Subsequent analyses revealed that there were significant differences in malevolent creativity performance among the three parenting styles, with participants in the *positive open parenting* having more malevolent creativity. Those with *undifferentiated parenting* had the lowest scores.

**Discussion:**

The findings provide theoretical guidance for parenting strategies. Future intervention studies on malevolent creativity should also consider the potential impact of parenting style to obtain better results.

## 1 Introduction

Creativity is widely acknowledged as a crucial faculty for navigating the myriad challenges and threats posed by our increasingly complex and ever-evolving environment. It is delineated as the process through which ideas, solutions, and products that are both novel and practical are generated (Amabile, 1982; Oldham and Cummings, 1996). From the utilization of innovative tools in early societies to enhance agricultural production to the reshaping of business transaction models through internet technology in contemporary society, creativity has always been a key driver for organizational growth and societal prosperity. It plays a significant role in artistic creation, medical breakthroughs, and in providing new strategies and tools for social development (Hermanni, 2016). While creativity is largely viewed as benevolent and positive due to its substantial contributions to societal progress and economic development, this perspective often overlooks that much creativity actually serves negative outcomes, such as the development of bullets and nuclear weapons, which cause immense harm and negative impacts on others and society (McLaren, 1993; Cropley, 2010). Just as a coin has two sides, creativity too possesses a “dark side.” When creative potential is utilized within a societal context to produce something deemed both novel and useful, with the intention of harming others or oneself for illicit gain, such acts of creativity are referred to as malevolent creativity (Plucker et al., 2004; Harris et al., 2013). The manifestations of malevolent creativity range from minor unethical behaviors such as lying to major criminal acts like terrorism and financial fraud, all of which can cause harm to individuals (Hunter and Cushenbery, 2015). Consequently, researchers have endeavored to investigate the elements that hinder malevolent innovation to limit or reduce its harmful consequences effectively.

According to the 6P model, the manifestation of malevolent creativity is influenced by six factors: process, person properties, person motivation, person feelings, product, and press (Cropley et al., 2008). However, not all these factors are conducive to the generation of malevolent creative ideas; it is only the specific combination of certain factors that facilitates malevolent creativity. The generative process of malevolent creativity typically depends on both internal personal factors and external environmental influences. Personal factors include traits such as sensitivity to aggression, anger, low emotional regulation, and low self-control, which can lead to the generation of more malevolent and harmful ideas in response to conflictual environments and problem-solving in daily life. Experiments have found that under conditions of low provocation and alcohol consumption, trait anger significantly predicts reactive aggressive behaviors within the Taylor Aggression Paradigm (Giancola et al., 2022). External environmental factors include social exclusion, unfair conditions, and provocation, which can spur more original creative ideas. Research using a modified version of the Prisoner’s Dilemma Task to manipulate social threats and the Alternate Use Task (AUT) for creativity assessment showed that social threats increased cognitive aggression and malevolent creativity, with participants generating more malevolent creative ideas compared to the control group (Harris et al., 2013; Harris and Reiter-Palmon, 2015; da Costa et al., 2020; Cheng et al., 2021). Despite these advances, few studies have discussed the mechanisms behind these different effects of malevolent creativity. Malevolent creativity shares cognitive foundations with general creativity but is distinguished by its malevolent intent and harmful characteristics. The generation of malevolent creative ideas and subsequent behavioral decisions remain influenced by cognitive reappraisal and emotional regulation, which can prevent the transformation from general to malevolent creativity. These capabilities are closely linked to an individual’s upbringing and experiences. Brooks (2001) suggests that future research should focus on parenting style. This not only helps children experience happiness, academic success, and life satisfaction and build a complete and healthy personality and quality but also improves their ability to cope with challenges and stress, such as self-control and emotional intelligence (Goldstein and Brooks, 2023). Following these expectations, this study focuses on the impact of parenting style on malevolent creativity and the underlying mechanisms (Wang, 2023).

Regarding the definition of parenting style, scholars propose that the more stable external behavioral patterns and styles displayed by parents in teaching and nurturing their children in their daily lives are a collection of more fixed attitudinal stances that are linked to rearing (Baumrind, 1971; Prevatt, 2003). In general, positive interaction between parents and children has a protective effect on the emergence and development of malevolent creativity. For example, good positive interaction styles and giving the child independent space to play can increase creativity beliefs and creativity self-efficacy, whereas behaviors such as excessive demands and imposing one’s will on the child are detrimental to the child’s creativity development (Moltafet et al., 2018; Gralewski and Jankowska, 2020). Positive, emotionally supportive parenting that creates a warm environment contributes to the formation of creative thinking in children, whereas disorganized, indifferent, emotionally neglectful parenting is harmful to creativity (Zhao and Yang, 2021). Childhood neglect as well as witnessing intimate partner violence are both risk factors for malevolent creativity (Mehrinejad et al., 2015; Jia et al., 2020). Previous research on the relationship between parenting styles and malicious creativity has primarily used a variable-centered approach focusing on specific parenting styles as predictors of malevolent creative behavior. However, parenting styles are multifaceted, and using a variable-centered approach that examines parenting styles separately would overlook the effects of various combinations of parenting styles on malevolent creativity (Fang and Shen, 2021; Li et al., 2022; Ren et al., 2022; Bedu-Addo et al., 2023; Shi et al., 2023). Based on the three types of parenting styles found in previous studies—positive, mixed, and negative parenting—we hypothesized that three types of parenting styles may exist (Qiu et al., 2022).

*H1*: There are three types of parenting styles.

Additionally, children who experienced parental neglect and apathetic rejection were more likely to have psychological disorders and social adjustment problems, exhibiting higher levels of aggression and anti-social problems (Wang, 2023). We hypothesized that participants with parenting styles similar to negative parenting styles may exhibit higher levels of malevolent creativity. Conversely, children with positive parenting styles may exhibit lower malevolent creativity, and emotionally warm family support may shape a child’s complete and healthy personality with higher self-control and empathy (Vasiou et al., 2023).

*H2*: Negative parenting styles may exhibit higher levels of malevolent creativity.

## 2 Methods

### 2.1 Samples and procedures

This study was reviewed and approved by the Ethics Committee of the Department of Psychology at Southwestern University. The questionnaire survey method was adopted by online distribution; 805 questionnaires were distributed to college students through an online network platform; 185 questionnaires that were not answered seriously, invalid, or with obvious patterns were excluded through two panic questions, and 620 effective questionnaires were retained. The age range of the participants was 15–30 years old (*M* = 21.04, *SD* = 2.41). A sample size of 620 is statistically sufficient to detect the correct number of profiles in LPA (Latent Profile Analysis) (Tein et al., 2013).

### 2.2 Measurements

#### 2.2.1 Parenting styles

We used a Chinese short-form of the Egna Minnen Beträffande Uppfostran: One’s Memories of Upbringing (s-EMBU-C). The scale has 42 items and consists of two main dimensions, father and mother, and each of these dimensions includes rejection, emotional warmth, and overprotection, for a total of six dimensions (e.g., “My father/mother wanted to decide how I should be dressed or how I should look”) (Li et al., 2012). In this study, father rejection, emotional warmth, and overprotection had a Cronbach’s α of 0.890, 0.893, and 0.784, respectively, while mother’s rejection, emotional warmth, and overprotection had a Cronbach’s *α* of 0.887, 0.886, and 0.815, respectively. The confirmatory factor analysis showed a good fit: *χ*^2^/df = 3.174, CFI = 0.852, TLI = 0.841, and RMSEA = 0.059.

#### 2.2.2 Malevolent creativity

We used the Malevolent Creativity Behavior Scale (MCBS) for measurement (Hao et al., 2016). The scale includes an array of 13 items (e.g., “How often do you have ideas about new ways to punish people?”), requesting subjects to evaluate the frequency of malevolent behavior they face in everyday life. The Cronbach’s *α* coefficient was 0.942, and a confirmatory factor analysis showed a good fit: *χ*^2^/df = 2.102, CFI = 0.981, TLI = 0.976, RMSEA = 0.042.

## 3 Results

**Figure 1:**
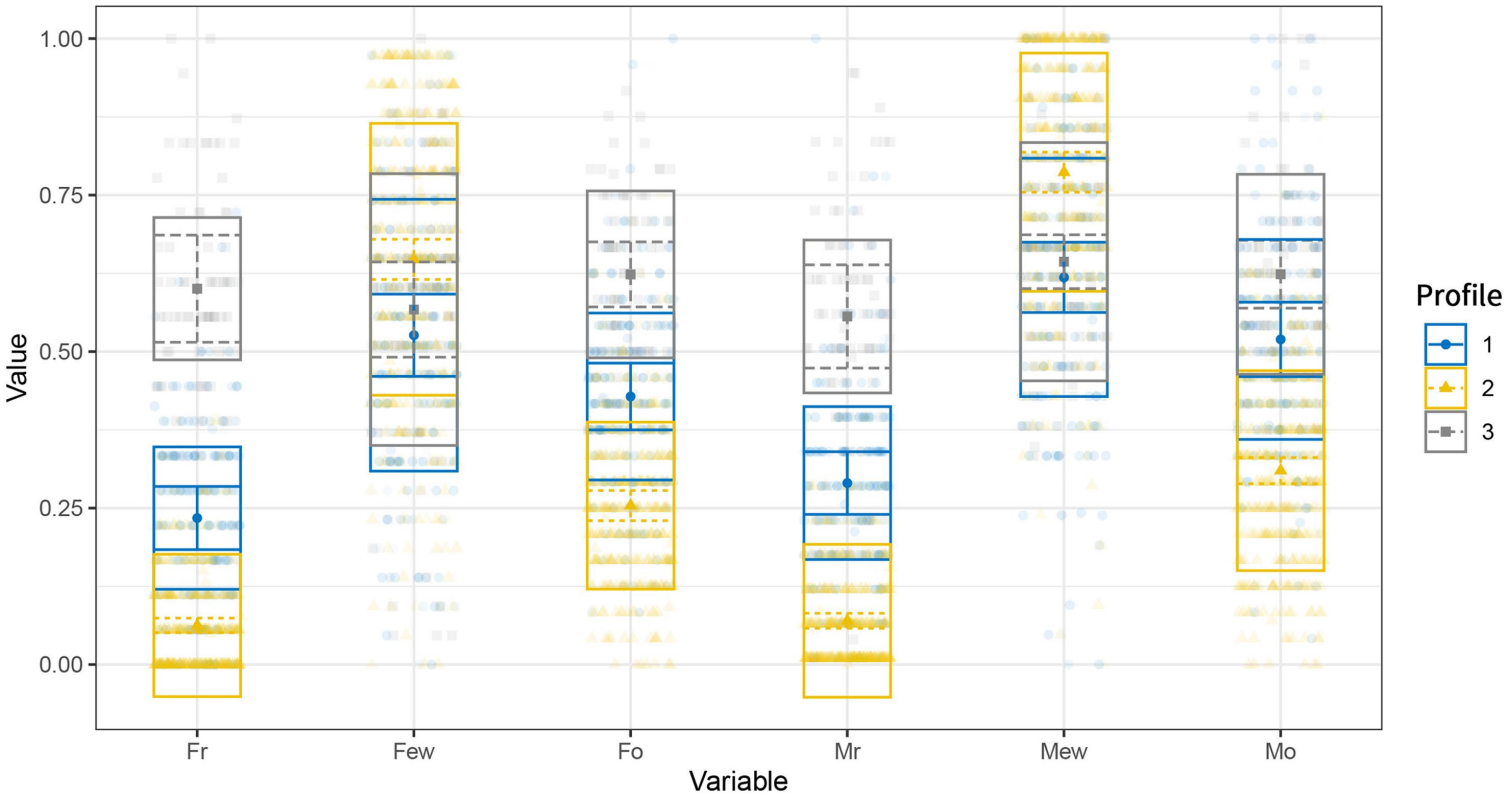
Description of LPA profiles. Fr, father’s rejection; Few, father’s emotional warm; Fo, father’s overprotection; Mr., mother’s rejection; Mew, mother’s emotional warmth; Mo, mother’s overprotection.

We completed the data analysis process using Mplus software and the R package tidyLPA (Rosenberg et al., 2019). We started with two profile models as the base model, added one profile in each turn, and picked the best model based on the fit index. After identifying the most appropriate model, we utilized the BCH method for a more profound examination of the possible differences in malevolent creativity (Asparouhov and Muthén, 2014). Table 1 describes the participants’ basic information and variable scores. Table 2 presents the values of the fit indicators for each LPA model. We considered a comprehensive range of indicators, and we selected the 3-profile solution as the best model. Figure 1 and Table 3 illustrate that Profile 1 had an average level on all indicators, suggesting that the participants may be in a balanced mode between positive and negative parenting styles. Therefore, Profile 1 is named *undifferentiated parenting*. Profile 2 had higher scores on the dimension of emotional warmth and lower scores on the dimensions of rejection and overprotection. Profile 2 was more prominent in the positive parenting style, which is labeled *positive open parenting*. The characteristics of Profile 3 are the opposite of the characteristics of Profile 1, with higher scores on the dimension of negative parenting styles. Profile 3 was named *negative limited parenting*. With this three-step approach, we further examined the differences in malevolent creativity performance among the subject populations with different profiles. As presented in Table 4, significant differences existed between the three parenting styles (*χ*^2^ = 254.61, *p* < 0.001). Children under *positive open parenting* characterized by high emotional warmth and low parental rejection and overprotection exhibited the highest malevolent creativity performance. A significant difference between *undifferentiated parenting* (*χ*^2^ = 231.20, *p* < 0.001) and *negative limited parenting* (*χ*^2^ = 64.62, *p* < 0.001) existed; the latter has the lowest malevolent creativity performance.

**Table 1:** Descriptive statistics (*n* = 620).

		*N*	%/*M* ± *SD*
Gender	Boy	244	39.4%
	Girl	376	60.6%
Birthplace	Townships	416	67.1%
	Countryside	204	32.9%
Father education	Primary education	54	8.7%
	Junior high school education	158	25.5%
	High school/secondary education	209	33.7%
	Post-secondary education	120	19.4%
	Bachelor’s degree or above	79	12.7%
Mother education	Primary education	75	12.1%
	Junior high school education	195	31.5%
	High school/secondary education	179	28.9%
	Post-secondary education	97	15.6%
	Bachelor’s degree or above	74	11.9%
Father’s rejection			1.53 ± 0.625
Father’s emotional warm			2.86 ± 0.691
Father’s overprotection			2.04 ± 0.554
Mother’s rejection			1.54 ± 0.625
Mother’s emotional warmth			3.17 ± 0.619
Mother’s overprotection			2.22 ± 0.603
Malevolent creativity			2.22 ± 0.876

**Table 2:** Fit indices and profile proportions for 2 ~ 5 profile models.

Profile	LL	AIC	BIC	SABIC	LMRT *p-*value	BLRT *p-*value	Entropy	Mixing ratio
2	−2991.04	6020.08	6104.25	6043.93	<0.001	<0.001	0.90	0.76/0.24
3	−2845.80	5743.60	5858.77	5776.23	<0.05	<0.001	0.86	0.61/0.12/0.27
4	−2773.94	5613.87	5760.05	5655.28	>0.05	<0.001	0.79	0.20/0.44/0.23/0.12
5	−2703.19	5486.38	5663.57	5536.58	<0.05	<0.001	0.83	0.01/0.23/0.41/0.23/0.11

**Table 3:** Profile 1 ~ 3 differences.

Profile	Fr	Few	Fo	Mr	Mew	Mo
Profile 1	1.189 ± 0.340	2.995 ± 0.669	1.764 ± 0.400	1.182 ± 0.370	3.358 ± 0.571	1.932 ± 0.480
Profile 2	2.805 ± 0.340	2.749 ± 0.669	2.872 ± 0.400	2.655 ± 0.370	2.932 ± 0.571	2.870 ± 0.480
Profile 3	1.706 ± 0.340	2.620 ± 0.669	2.286 ± 0.400	1.853 ± 0.370	2.853 ± 0.571	2.561 ± 0.480

Fr, father’s rejection; Few, father’s emotional warm; Fo, father’s overprotection; Mr, mother’s rejection; Mew, mother’s emotional warmth; Mo, mother’s overprotection.

**Table 4:** Profile differences on malevolent creativity.

	Undifferentiated parenting	Positive open parenting	Negative limited parenting	
	Profile 1 (*n* = 381)	Profile 2 (*n* = 73)	Profile 3 (*n* = 166)	Approximate *χ*^2^
	*M(SE)*	*M(SE)*	*M(SE)*	
Malevolent creativity	1.86(0.04)^c^	3.44(0.10)^a^	2.46(0.07)^b^	254.61

Lowercase a is significantly higher than lowercase b and lowercase c, while lowercase b is significantly higher than c. And three different lowercase letters, abc, mean there are significant differences between them.

## 4 Discussion

This study used a person-centered approach (LPA) to examine the association between different parenting style patterns and malevolent creativity performance. The results of the LPA indicate three different profiles of parenting styles: undifferentiated parenting (medium level of engagement and dysfunctional), positive open parenting (high level of emotional warmth and supportive openness), and *negative limited parenting* (high level of dysfunctional and limited).

The subsequent analyses indicated that three different parenting styles exhibited significant differences in malevolent creativity. These three parenting styles were similar to the results of previous studies: positive parenting, negative parenting, and moderate parenting. Particularly, positive parenting was consistent with *positive open parenting* characteristics, representing high levels of emotional warmth (Qiu et al., 2022). Previous research has also classified parenting styles as permissive, typical, and authoritarian, with authoritarian parenting similar to negative limited parenting (Klukas et al., 2021). These studies not only indicate the accuracy of the three profiles we use but also emphasize the stability of the parenting style categories. In a further study, we found that positively open-parented adolescents exhibit higher levels of malevolent creativity, which differs from the previous study (Wang, 2023). We propose the following reasons: (1) Malevolent creativity belongs to the creativity category; the core of malevolent creativity is to generate novel and malevolent creative ideas, which remain influenced by working memory and cognitive flexibility factors. It has been demonstrated that warm parental support is positively correlated with children’s ideational fluency, flexibility, and originality (Harvey and Berry, 2022). It dramatically improves children’s working memory and executive functioning (Ren et al., 2017; Lam et al., 2018). Overprotective parents who impose their ideas on their children neglect their needs for autonomy and self-esteem. This prevents them from facing challenges and difficulties independently, which are essential preconditions for creativity (Pinquart and Gerke, 2019). However, a friendly and positive atmosphere in which parents interact with their children, giving them more freedom to use their imagination, enhances their creative motivation to produce more original ideas (Ryan and Deci, 2000; Gralewski and Jankowska, 2020). (2) Lower parental rejection and parental overprotection may lead to the phenomenon of spoiling and indulging children’s immoral behaviors, failing to stop their initial malicious behaviors, which causes a lack of moral development in children (Ścigała et al., 2022). This makes them more likely to engage in violent and antisocial behaviors when they mature (Schroeder et al., 2010). Here, our results differed from previous research (Csathó and Birkás, 2018). Children with intact and healthy family relationships exhibited more malevolent creativity performance when compared to those children who were not thoughtfully cared for by their parents. Therefore, we propose that parents should provide warm emotional support, create harmonious and positive interactions with their children, and emphasize their moral development by immediately providing them with necessary and preventative education when they engage in unethical behaviors (Fatima et al., 2022; Luo and Bussey, 2023).

First, we supplemented the malevolent creativity component of the effect of family parenting styles on the development of creative thinking. We obtained more accurate results using latent profile analysis by linking the combined combination of specific parenting styles to malevolent creativity performance. Second, our study can also provide some help for parents’ parenting strategies and parenting models: for the good development of their children’s creative thinking, parents should pay more attention to the development of their children’s moral cognitive development while providing positive emotional warmth and a good atmosphere for parent–child interactions and should add more moral elements into their normal educational environment to help their children’s growth.

There are a few limitations to this study that deserve clarification. First, the data and sample were distributed online and limited to a single university, making it challenging to ensure the validity of the data and extend the results to other cultures and sample populations. Future studies could consider large, diverse, and multicultural sample populations to help draw generalizable conclusions. Second, although we used latent profile analysis to examine the data, we could not establish a definitive causal relationship between parenting style and malevolent creativity. In future research, we encourage using experimental methods or longitudinal studies to explore the effects of family parenting styles on the fluency, novelty, or flexibility of malevolent creativity and its associated influence factors, such as family moral education.

## 5 Conclusion

We used latent profile analysis to classify three parenting styles in a sample of college students. The results indicated that significant differences exist in malevolent creativity performance among college students in the three parenting modes, with subjects in the *positive open parenting* mode exhibiting more malevolent creativity. Therefore, we propose that parents should focus on the development of their children’s moral character while providing warm emotional support.

## Data availability statement

The raw data supporting the conclusions of this article will be made available by the authors, without undue reservation.

## Ethics statement

The studies involving humans were approved by Ethics Committee of the Department of Psychology at Southwestern University. The studies were conducted in accordance with the local legislation and institutional requirements. Written informed consent for participation was not required from the participants or the participants’ legal guardians/next of kin in accordance with the national legislation and institutional requirements.

## Author contributions

XC: Writing – original draft. XZ: Writing – review & editing. HZ: Writing – review & editing, Data curation.
